# Roles and Prospects of Dengue Virus Non-structural Proteins as Antiviral Targets: An Easy Digest

**DOI:** 10.21315/mjms2018.25.5.2

**Published:** 2018-10-30

**Authors:** Hannah Norazharuddin, Ngit Shin Lai

**Affiliations:** Institute for Research in Molecular Medicine (INFORMM), Universiti Sains Malaysia, 11800 USM, Pulau Pinang, Malaysia

**Keywords:** dengue virus, non-structural proteins, antiviral target

## Abstract

Dengue is a neglected disease caused by the infection of dengue virus which is transmitted by *Aedes* mosquitoes and to some, it could be fatal. Regardless of the enormous work devoted to research for the treatment of dengue, to this day there is no cure, and treatment is solely limited to supportive care by treating the symptoms. The inhibition of the viral RNA non-structural enzymes has been the most popular approach amongst the strategies applied to the search and development of dengue antivirals. This review is a compact digest of what is already known of the roles and the prospects of the dengue virus non-structural proteins NS1, NS2BNS3, NS4A, NS4B and NS5 as the targets for antiviral studies including the recent progress that has been published regarding their roles.

## Introduction

The Dengue Virus (DENV) is an arthropodborne virus (arbovirus) of the *Flaviviridae* genus which also includes over 70 other important human pathogens such as Yellow Tick-Borne Encephalitis Virus (TBEV) (1). DENV is grouped into five serotypes, and these serotypes are antigenically distinct although closely related (DENV1, DENV2, DENV3, DENV4 and DENV5) with the fifth serotype recently discovered in late 2013 (2). Infection of this virus across different serotypes causes a range of illness extending from unapparent febrile illness, which often is diagnosed as Dengue Fever (DF) to an acute and potentially lethal hemorrhagic fever known as Dengue Hemorrhagic Fever (DHF) (3). The lifelong immunity provided by the recovery from the infection with one of the DENV serotypes is specifically against that particular serotype, but cross-immunity to the other serotypes is only partial and temporary. The transmitter mosquito *Aedes* is found in tropic and sub-tropic regions of the world, which includes parts of Indonesian archipelago into Northeastern Australia, South and Central America, Southeast Asia, Sub-Saharan Africa and some parts of the Caribbean. The occurrence of the disease has grown at an alarming rate around the world in recent decades with an estimation of 50–100 million infections worldwide every year (4).

Efforts to develop a vaccine for DENV have been in the works since in the early 1920s. To date, there are a number of DENV vaccine candidates being developed and live attenuated vaccine candidates are the furthest in the development pipeline with a total of six in the clinical development stage (5, 6), such as TV003/ TV005 by the US National Institutes of Health and Butantan, DENVax by Takeda and DEN-80E by Merck to name a few. One finally made its way to the public after being approved in several countries according to the World Health Organization. The recently licensed vaccine is a tetravalent vaccine, appropriately named Dengvaxia^®^, developed by the pharmaceutical giant Sanofi Pasteur (6). Despite the excitement over the vaccine, Malaysia has yet to make its decision on whether or not to roll out the vaccine for public use questioning its efficacy and economic impact (7). The question of the vaccine efficacy is highlighted as it shows variation by the recipient’s age and serostatus, and also by the DENV serotype causing the infection with higher efficacy is witnessed in DENV 3 and 4 compared to DENV 1 and 2 (8).

As of now, there is no anti-viral drug that has been successfully developed albeit the increasing need. The current treatment is merely focused on treating the symptoms relying entirely on supportive care (9). Anti-viral approaches have explored structural and non-structural proteins of DENV as targets. The usage of therapeutic antibodies is the most advanced intervention against virus entry although small molecules have been examined. The enzyme NS3 and NS5, along with NS4B and C protein, are the main focus of the search for small-molecule inhibitors. Studies have identified a number of compounds that can be successfully used as inhibitors (Table 1). Nevertheless, the only clinically investigated drug which is believed to directly target the viral protein NS5 is balapiravir, which is a nucleoside analogue originally developed for hepatitis C. However, a clinical trial showed that the drug did not meet efficacy endpoint (10).

## DENV Life Cycle and Protein Processing

Flaviviruses are icosahedral in shape and encase a single-stranded, ~11 kb, positive-sense RNA genome (Figure 1) within its capsid protein in a host-derived lipid bilayer. Mature DENV virions consist of three structural proteins, the capsid protein (C), membrane protein (M), and the envelope protein (E). The C protein (11 kDa), in multiple copies, encapsulate the RNA genome forming the viral nucleocapsid. The nucleocapsid is surrounded by a host cell-derived lipid bilayer, in which 180 copies of M and E are anchored. The M protein is a small (~8 kDa) proteolytic fragment of its precursor form prM (~21 kDa). The E protein is sized at 53 kDa and has three distinct structural domains (21).

The virus enters the host via a process of receptor mediated-endocytosis followed by fusion of the viral and vesicular membrane, allowing the release of the genomic RNA into the cytoplasm, serving as mRNA for replication and translation. The viral RNA carries an open reading frame (ORF) encoding a single polyprotein that is translated in a cap-dependent manner in the endoplasmic reticulum (ER) (22). The large viral polyprotein is co-translationally and post-translationally processed into three structural proteins (C, prM, and E) located at the N-terminal, and seven non-structural (NS) proteins (NS1, NS2A, NS2B, NS3, NS4A, NS4B, and NS5) are encoded at the C-terminal. The viral proteins prM, E, NS1, and NS4B are generated in the host’s ER Lumen by utilising the host’s ER signal peptidase which constructs the amino termini of prM, E, NS1, and NS4B by cleavage. The other NS proteins and the C-terminus of the C protein are processed by the viral two-component protease NS2B-NS3 in the cytoplasm of the infected host. At a later stage of infection, a Golgi localised furin protease generates mature membrane protein M from its precursor prM (23).

## NS3 Helicase

The protein NS3 of DENV is the second largest non-structural protein (~70 kDa) of the virus, and it has been found to exert numerous enzymatic activities during the virus replication. The N-terminal region forms a serine protease needed for the polyprotein-processing together with the protein NS2B as a co-factor. On the C-terminal of the protein, NS3 contains a nucleoside triphosphatase (NTPase), a 5′ RNA-triphosphatase (RTP) and helicase in the remaining 70% of the protein (24). The helicase is essential for DENV replication process, and this has been shown by mutagenesis of the corresponding domain (25). The importance of NS3 protein for the survival of the virus makes it a suited target to be studied extensively as a drug target.

The helicase domain of NS3 (residues 180–618) comprises three sub-domains with structural and significant sequence identity resemblance to the helicases of another flavivirus (26). The ATPase/helicase and NTPase activities of DENV NS3 share the same active site and both C-terminal domain activities combined are required for melting secondary structures prior to initiation of RNA synthesis and for the unwinding of RNA duplexes, either to separate double-stranded RNA (dsRNA) intermediates formed during viral RNA synthesis or as a translocase that can remove proteins bound to viral RNA (27–29).

It was previously suggested that the protease domain exert significant regulatory role on flavivirus NS3 helicase activity due to an overlap region of 20 amino acids (29, 30). Despite the strong suggestions, the NS3 protease domain does not modulate the ATPase and helicase activities of DENV NS3. The helicase domain, encompassing amino acids 171 to 618, has steady-state kinetic properties similar to that observed for the full-length NS3 with no critical differences between the two enzymes regarding RNA allosteric activation of ATP hydrolysis and RNA unwinding time courses (31). The virus loses its ability to replicate when its helicase activities are diminished, confirming the importance of the function of NS3 helicase domain in the continuation of the viral life cycle (25), and this prompts for development of the inhibitors or modulators of this enzyme as therapeutic agents (30).

A comprehensive list of helicase inhibitors listed six compounds as the inhibitor for flavivirus replication (32). Among them is Ivermectin. In silico docking of compounds into the single-strand RNA access site of WNV helicase identified Ivermectin which inhibited WNV, YFV, and DENV helicase dsRNA unwinding activity but not the NS3 ATPase activity. When tested in cell culture, ivermectin, that has been shown previously to inhibit DENV protease activity (15), inhibited YFV as well as DENV, JEV, WNV, and TBEV but with less potency (16). Ivermectin is a broadly used anti-helminthic drug and has been in clinical use for 25 years, yet it remains to be determined whether it would be efficacious with DENV. Another potential candidate for drug development is the small molecule ST-610 which potently inhibited all four serotypes of DENV in cell culture (17). ST-610 inhibits DENV helicase RNA unwinding activity, but not ATPase activity. ST-610 showed marginal efficacy in the DENV AG-129 mouse model. The in vivo pharmacokinetic properties of this compound have much to be improved for further development.

## NS3 Protease (NS2BH-NS3pro)

The DENV NS3 protease (NS3pro) is a trypsin-like serine protease that accommodates a typical serine protease catalytic triad comprising of the residues histidine 51 (His51), aspartic acid 75 (Asp75), and serine 135 (Ser135) located within 180 amino acids in the N-terminal one-third of the protein (33, 34, 35). The catalytic activity of the protease enzyme requires the central ~40 residue hydrophilic domain from NS2B to function effectively (NS2BH-NS3pro) (36, 37). NS2BH-NS3pro only recognise sites with two cationic residues, unlike the conventional trypsin which recognises sites containing a single cationic residue (38). The protease cleaves the following sites: i) NS2A-NS2B, NS2B-NS3, NS3-NS4A, NS4B-NS5, ii) upstream of signal sequences at the C-prM and NS4A-NS4B junctions, iii) within NS2A, and also iv) within NS3 itself (39, 40).

With the need of both domains in order to function, NS2BH-NS3pro has been expressed by covalently linking the C-terminus of NS2BH cofactor to the N-terminus of NS3pro through a flexible 9-residue Gly4SerGly4 linker (41); and a more recent expression system of simultaneous uncoupled expression of NS2BH-NS3pro without the presence of a linker has been reported to dismiss the arguments of unknown effect of the artificial linker introduced between the protease and its cofactor that provide a more realistic model for screening inhibitors (42). The enzyme’s substrate processing activity has shown to be affected by product release, suggesting a difference in conformation of the product-bound enzyme to the substrate-binding conformation and providing an important clue for alternative inhibitor design (42).

The engineering of the binding loop of aprotinin, a serine protease inhibitor which is potent against DENV NS3 protease (41, 43) shows that the prime side significantly modulates DENV protease binding affinity which could solve the hydrophilic and non-specific scaffolds resulting from the non-prime side. The tightest cyclic peptide achieved a Ki value of 2.9 μM against DENV3 WT protease by optimising the cyclisation linker, length, and amino acid sequence of a series of cyclic peptides. This provides proof that both sides of the DENV protease active site have the potential to be explored in order to achieve specificity and lower hydrophilicity for the design of DENV inhibitors (44).

The DENV protease functions in a highly similar way to its HCV counterpart. Unlike HCV, proteases from the four serotypes of DENV share similar substrate specificity and higher sequence similarity (45), imply that it is possible to develop a single inhibitory agent targeting all four dengue NS3 proteases. A benzimidazole derivative, named MB21, was found to be highly potent in inhibiting NS2BH-NS3pro (IC50 = 5.95 μM) and was also found to be effective in inhibiting each one of the four serotypes of DENV in infected cells in culture, based on analysis of viral antigen synthesis and infectious virus production without showing any distinguishable cytotoxicity (46).

## NS5

The NS5 protein is the largest and most conserved flaviviral protein of about 900 amino acids long and has the molecular mass of about 100 kDa which harbours an RNA-dependent RNA polymerase (RdRp) domain at its C-terminal end, which is immensely vital for the viral replication (24, 47). The large protein also contains a Capping enzyme site, which is made up of a methyltransferase (MTase) and guanylyltransferase (GTase) domain at its N terminus (48–50).

The NS5 capping enzyme (CE) has critical involvements in the mRNA capping process due to its methyltransferase (MTase) and guanylyltransferase (GTase) activities (33). The N-terminal of the NS5 CE is responsible for transferring a guanosine monophosphate (GMP) from guanosine triphosphate (GTP) to the diphosphorylated RNA and for adding methyl groups to the guanine N-7 and ribose 2′ hydroxyl positions of the viral cap (51, 52).

Despite playing a central role in viral replication in the cytoplasm of infected host cells, the NS5 protein is located pre-dominantly in the nucleus of infected cells, which leads to the belief that it plays a role in the suppression of the host anti-viral response (53). The enzymatic activities of NS5 indicate the enzyme’s standing role in the replication of the virus, playing a decisive role to preserve the virus longevity and survival, prompting NS5 as a promising anti-viral target (11, 54).

## NS1

DENV non-structural protein 1 (NS1) is a glycoprotein with a molecular weight ranging from 46 to 55 kDa due to its degree of glycosylation (55). The protein is expressed as a monomer, a membrane-bound dimer on the cell surface and secreted as a hexamer into the blood circulation of patients which forms an open barrel structure with three dimers forming a hydrophobic central cavity carrying ~70 lipid molecules (56). The dimeric NS1 plays a pivotal role in the viral genome replication likely through interactions with NS4A and NS4B transmembrane proteins in the early stages of infection (57, 58).

Unlike the rest of the non-structural proteins, NS1 is noteworthy for being the target of dengue fever diagnostic kits. One rapid test is an Elisa based Platelia Dengue NS1 Ag assay developed by Bio-Rad in partnership with Pasteur Institute in France. The high levels of NS1 detected in patient’s sera allows for the early diagnosis of dengue fever which drove the development of dengue diagnostic tests (59). The association of the NS1 antigen positivity with a higher risk of advancing to severe dengue can potentially be used as a test for the sign of severe dengue (60, 61).

The NS1 in hexameric form has long been hypothesised to play a role in dengue physiopathology due to its content of triglycerides, cholesterol, and phospholipids which is also present in high-density lipoproteins (HDL) suggesting that NS1 transports lipids in the plasma from tissues to the liver of dengue patients (62).

Recent findings have uncovered that NS1 activates macrophages via Toll-like receptor 4 (TLR4) and disrupts endothelial cells which cause vascular leakage, a distinct characteristic of dengue hemorrhagic fever (DHF) and dengue shock syndrome (DSS) (63, 64). This action is similar to the mechanism of septic shock in bacterial infections caused by bacterial cell wall products, indicating NS1 protein to be a viral toxin. This opens up a whole new avenue in repurposing clinically available drugs for dengue fever treatment.

## NS4A/B

There is still more to be understood about the functions of the transmembrane proteins NS4A and NS4B. Instead of harbouring an enzymatic activity, these proteins act as scaffolds for the replication complex formation (57, 65). A transmembrane peptide named 2K connects the two non-structural proteins together; it is then cleaved during the polyprotein maturation. NS4A has shown to induce the re-arrangement of the endoplasmic reticulum (ER) (66). NS4A also induces the localisation of the replication complex to be stabilised in the perinuclear area. This localisation is important due to the need for RNA viruses to use nuclear components for replication (67). In DENV infection, the stabilisation of the replication complex is mediated by the interaction of NS4A with the vimentin scaffold (68), which is a component of intermediary filaments for vesicular/organelle positioning and transport.

NS4B modulates the viral replication by its interaction with the helicase domain of NS3 to assist its dissociation from the viral RNA (69). Rather than having direct involvement with the viral genome replication process, NS4A participates in viral replication by inducing autophagy, hence protecting the cells from cell death during infection which is vital to viral replication (70).

## Conclusion

To conclude, the DENV non-structural proteins play diverse yet vital roles in ensuring the survival of the virus. Abundant studies done on the DENV non-structural proteins have shown that they are promising anti-viral targets. New roles and functions of these proteins have been uncovered, and this opens up many possibilities to manoeuvre research into finding the best anti-viral target. With the current urgency in finding the best treatment for dengue supported by the hints provided by the research done by various groups, we have to keep on moving towards discovering a cure for dengue fever.

## Figures and Tables

**Figure 2:**
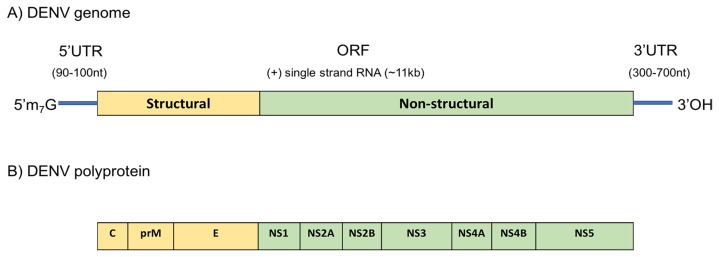
Structure of the DENV genome. A) The 5′ end is capped with N^7^methylated guanosine cap while the 3′ end forms a hairpin loop. The genome translates into B) a single polyprotein which will then be processed by the viral and host mechanism

**Table 1:** Prospective DENV anti-virals and their current status in drug development

Compound	Mode of action	Current status	Reference
Balapiravir	NS5 polymerase nucleoside analogue	Showed no support for balapiravir as a drug candidate	(10)
4-HPR	NS5 methyltransferase	Showed efficacy in a mouse model, tolerable human profile.	(11)
Retrocyclin 1	NS2BNS3 protease inhibitor	Laboratory; significantly reduced viral replication in DENV-2 infected Vero cells	(12)
BP13944	NS2BNS3 protease inhibitor	Laboratory; reduced DENV replicon reporter expression in cells, (EC50) of 1.03 ± 0.09 μM	(13)
ST-148	Capsid inhibitor	Preclinical development	(14)
ARDP0006, ARDP0009	NS2BNS3 protease inhibitor	Laboratory; showed inhibition of DENV-2 virus replication in cell culture.	(15)
Ivermectin	NS3 Helicase	Clinical trial estimated completion date on February 2016	(16)
ST-610	NS3 Helicase	Showed inhibition of all four DENV serotypes in cell culture	(17)
Suramin	NS3 Helicase	Potent NS3 helicase non-competitive inhibitor	(18)
AM404	NS4B	Showed inhibition of DENV replication	(19)
Lycorine	NS4B	Potent inhibitor for flavivirus in cell culture	(20)
